# Enabling Flexible and Continuous Capability Invocation in Mobile Prosumer Environments

**DOI:** 10.3390/s120708930

**Published:** 2012-06-28

**Authors:** Ramon Alcarria, Tomas Robles, Augusto Morales, Diego López-de-Ipiña, Unai Aguilera

**Affiliations:** 1 Department of Telematics Engineering, Technical University of Madrid, Avenida Complutense n. 30, Ciudad Universitaria, 28040 Madrid, Spain; E-Mails: trobles@dit.upm.es (T.R.); amorales@dit.upm.es (A.M.); 2 Deusto Institute of Technology, University of Deusto, Avda. Universidades 24, 48007 Bilbao, Spain; E-Mails: dipina@deusto.es (D.L.-I.); unai.aguilera@deusto.es (U.A.)

**Keywords:** prosumer, software engineering, ubiquitous computing, communication paradigms, resource management

## Abstract

Mobile prosumer environments require the communication with heterogeneous devices during the execution of mobile services. These environments integrate sensors, actuators and smart devices, whose availability continuously changes. The aim of this paper is to design a reference architecture for implementing a model for continuous service execution and access to capabilities, *i.e.*, the functionalities provided by these devices. The defined architecture follows a set of software engineering patterns and includes some communication paradigms to cope with the heterogeneity of sensors, actuators, controllers and other devices in the environment. In addition, we stress the importance of the flexibility in capability invocation by allowing the communication middleware to select the access technology and change the communication paradigm when dealing with smart devices, and by describing and evaluating two algorithms for resource access management.

## 1. Introduction

Uniform access to resources and devices is one of the most discussed topics in ubiquitous computing related work. This is demonstrated by the large amount of work on communication middleware available today [1,2]. Continuous service execution requires addressing device interoperability problems, due to the wide heterogeneous nature of existing devices. The most common interoperability issues are related either to technology and access protocols or the format of the exchanged data, that is, the set of rules that must be taken into account to interpret the data once obtained. This work deals with the first category, which includes interoperability issues related to communication paradigms. Communication paradigms are often bounded to the employed protocol but sometimes they are decoupled. Connection-oriented, synchronous or asynchronous, message-based or service-based communications are considered in this category. This paper contributes to solve some of the issues related to communication middleware, regarding flexible and continuous service executions. For this, we describe the design and validation of a middleware solution for continuous capability access whilst service execution is taking place in ubiquitous environments. The main contribution of the paper is the definition of the communication middleware's architecture and its integration with other interdependent subsystems. Also, an implementation of this resource access middleware is proposed that integrates a set of access technologies and communication paradigms, which are commonly used and requested by available capabilities.

The presented middleware contributions are based on the work done in the mIO! project, which aims at the provision and consumption of prosumer services in a mobile environment. The term prosumer [3] (as an acronym formed by the fusion of the words producer and consumer) is applied to those users that are at the same time consumers and producers of services or contents. In our view, these users, placed in the center of device-rich environments, uses their smartphone to design, compose and configure new services with the help of creation tools. In mobile prosumer environments, the generated services use the available functionalities offered by surrounding devices and nearby elements. The middleware will try to guarantee that the service execution is maintained even though the used elements may change or disappear.

Mobile prosumer environments establish some requirements that determine the design of the proposed architecture. Focusing on non-expert users, a high level of abstraction is required in order to enable users to create their own services in an easy way. Besides, the architecture needs to adapt to changes in the availability of resources and services, as well as to provide a communication infrastructure for uniform resource access. For further information about the prosumer concept and the mIO! architecture the reader is referred to our previous work [4]. Based on the requirements of the mobile prosumer environment, the service must present a logical structure defined by different layers, described in the next Section. Section 3 analyses the communication paradigms currently used in communication middleware. Section 4 describes the overall architecture, which has been designed using various design patterns in order to meet the requirements imposed by the ubiquitous environment and the studied communication paradigms. Sections 5 and 6 describe the integration between the resolution and capability invocation processes while Sections 7 and 8 make a contribution to the communication with smart devices and the problem of resource and connection management respectively. Finally, the paper concludes with a validation of the designed system, related work and some conclusions of the proposed solution.

## 2. Service Logical Model

The provision of a higher level of abstraction for prosumer users leads to the following concepts: *Service*, as a unit supplied and consumed by the prosumer, *Component*, which represents a basic and functional unit used by a service, and *Capability*, which is the implementation of the functionality defined by a component and provided by some element (hardware or software). It is also necessary to introduce the concept of *Orchestration*, which manages the interaction between the different components of a service, *Resolution*, which manages the association of capabilities to components and, finally, *Invocation*, which provides a uniform access to the infrastructure capabilities.

**Figure 1.**
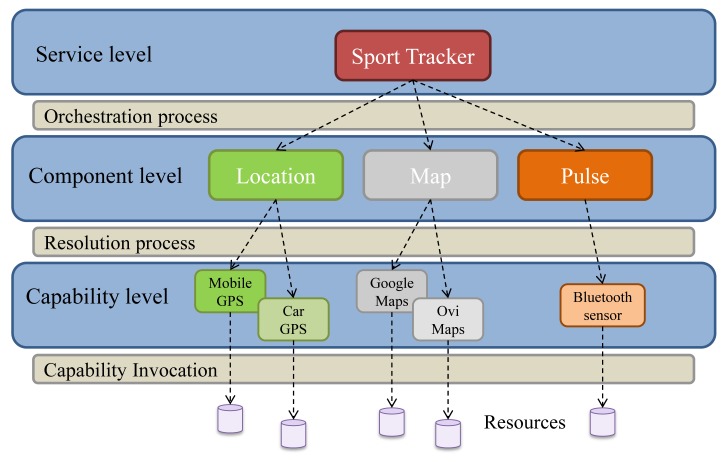
Service logical parts.

A service can be defined in many ways, depending on the state of the life cycle in which the service is. We define the logical structure of a service by different levels: the **service level**, the **component level** and the **capability level**. To illustrate these concepts we present the example of a simple prosumer service, called *Sport Tracker*, which aims to access the location information of a user and to represent it on a map along with information about his heartbeat. This service consists of three components: a *Map* provider, a *Location* provider and a *Pulse* provider. These abstract services provide an interface that must be implemented so that the composed service can be executed. For example, the *Location* component needs to be resolved into a GPS device or a GPS capability (e.g., offered by a mobile phone) in order to obtain the information about the user's location. Figure 1 shows the proposed service logical model, adapted to this simple example service.

The three-level model is explained below:

The first level is the **service level**, where services are seen as a software structure that can be provided and consumed in a mobile device, with the capability of performing tasks where the different components used internally remain hidden. Service behavior and orchestration logic are specified through a Service Description Language (SDL) document.

Continuing with the **component level**, the service is split into different logical units called components, which interact according to the logic defined by the service. A component is a basic and functional unit of a service. It is a high level functional abstraction that is implemented by a given capability depending on the service execution conditions. Components are used by the system to make the creation process easier and provide the adequate abstraction level so that a user can understand their functionality whereas the implementation details remain hidden. In order to make a step towards the new mobile prosumer environment, developers must implement and publish a big number of different components, which cover all the creation possibilities that a user could wish.

The **orchestration process** manages the interaction among components, *i.e.*, how the components are interconnected to compose services, how are the components managed and the data exchanged inside the architecture and how the components obtain the appropriate capability to implement their functionality. This process takes place during creation time and is performed by the creation subsystem.

Finally, in the **capability level** a service is seen as a set of capabilities which offer the functionalities that are demanded by the service. Capabilities generally access to local (in-device), nearby or remote resources and are designed to achieve the components' objectives. The division between the component and the capability models is made for two reasons. First, the orchestration logic is decoupled from the implementation. This way, a component can be resolved into different capabilities depending on the service execution conditions and the preferences given by users in the creation process. Second, components are defined as functionalities that are easy to understand for non-expert users in order to help them to create simple services.

The **resolution process** assigns, during execution time, each component to the best available capability that can implement it. This process takes place in the *harmonization subsystem*. Finding the optimal capability depends on multiple factors, for example, the configuration options established by the user during creation time or component requirements.

In the *Sport Tracker*'s example scenario, since there is only one compatible capability for the *Pulse* component, it can only be mapped to the Bluetooth sensor capability whereas the *Map* component can choose a map provider based on user preferences or service restrictions. A component, by this way, defines some requirements so that the election of the capability is the optimal one.

The **capability invocation process**, which is the main topic of this paper, is the one responsible for requesting and obtaining all the required resources. An effective coordination between the resolution and the invocation processes will enable continuous mobile service execution in dynamic environments. In these environments, the capabilities can appear and disappear at any time and the wide variety of sensors, actuators and other devices makes necessary to design different mechanisms for the access and invocation of heterogeneous capabilities. This process takes place in the Capability Middleware and is explained in section 4, along with the Creation, Execution and Harmonization subsystems.

## 3. Communication Paradigms in Capability Access

Most mobile middleware solutions for resource access include only one communication paradigm, ignoring the fact that device configurations and conditions in such environments are extremely varied. Therefore, we have designed a capability access middleware that includes several communication paradigms, classified under three criteria:

- *Coordination Mechanism*: Differentiates between synchronous or asynchronous communication models.
- *Notification Model*: determines if consumers explicitly retrieve new messages or are notified when new messages are produced (synchronous or asynchronous notification).
- *Connection Orientation*: many middleware platforms employ the notion of message as a fundamental building block (Message-oriented Middleware). Other middlewares use the concept of session to communicate with resources [5], providing channel and transaction management [6]. A connection oriented middleware uses sessions instead of single message interchange as the most natural method of communication.

The studied paradigms are described below. Table 1 shows the features of these paradigms according to defined criteria and the requirements that they impose on the design of system's architecture, presented in the next section.

### 

#### Request-Reply model

a synchronous model is adopted in situations that require the communicating entities to be connected simultaneously. The sending entity delegates the control to the receiving entity, which performs some processing and responds, allowing the first to continue its execution.

#### QP2P (Queue-based Point-to-point Paradigm)

distributed queues are used for sending and receiving messages. Using this model, messages are obtained in a predefined order based on queue type (FIFO, LIFO and so on). Producers and consumers are fully decoupled.

#### Tuple Spaces

this paradigm provides a distributed shared memory for the exchange of tuples between various entities, based on the Linda's model of communication [7]. Tuples are data structures that can be inserted, modified and removed from the shared space. Like QP2P, the Tuple Spaces paradigm uses an indirect model, mediated by a *Tuple Space Service*, but in this case the consumer gets messages (tuples) by requesting them directly to this service.

#### Publish-Subscribe

communicating entities exchange messages by publishing events and subscribing to them. Generally, an intermediate service called Event Channel [8] is introduced, which registers the subscriptions and forwards the events published. In pub-sub systems, message delivery depends fully on the actions of the receivers, which frequently are unknown to the senders.

## 4. Overall Architecture for Service Orchestration, Resolution and Invocation

**Figure 2.**
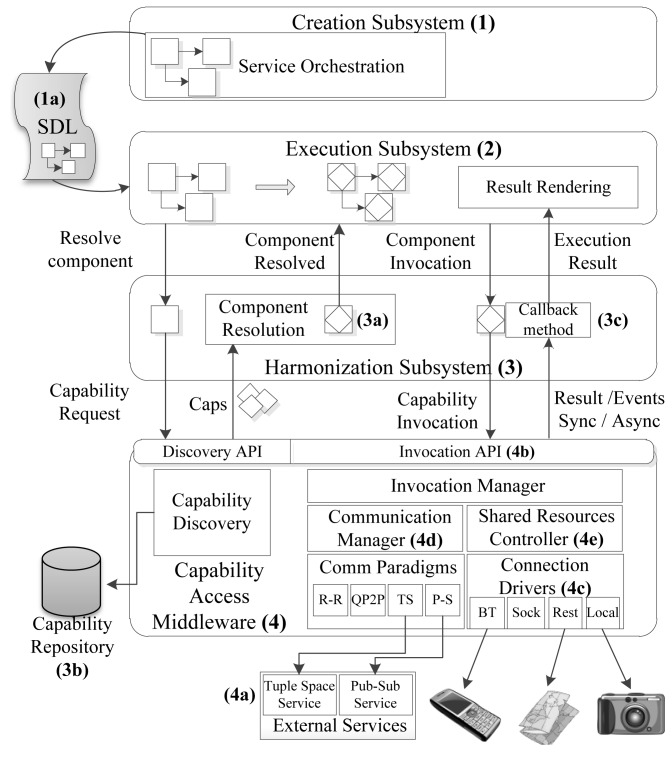
Overall architecture.

The service provision and consumption platform described in this paper is designed for the mobile device of the prosumer user, and consists of a set of subsystems (see Figure 2) which perform the functions of orchestration, resolution and capability invocation. The design of these subsystems is affected by the communication paradigms that address the capability access, which relate to the need for external services (services not included in the mobile phone) to manage deployed tuples and publication/subscription records (4a) and the environmental requirements for mobile prosumer users, stated in the introduction section.

Design patterns are used to address the requirements of the prosumer environment in an elegant and effective way [9]. The application of these patterns can impact the ability of systems to achieve their quality attribute goals, and, therefore, they affect the system architecture and help to address key issues that are resolved in the following sections. Table 2 shows some requirements from the prosumer environment, the associated pattern that has been chosen to deal with each requirement and the implications in the overall architecture. The proposed subsystems are:

### 

#### Creation Environment (1)

It provides mechanisms for service creation and composition by non-expert users through component interconnection and customization. Specifically, the user drags and drops some components into the creation environment and establishes some connections between them. This is possible because the platform provides a component repository in which components are published by external developers so that the creator can use them. The creator configures the components in order to set restrictions that will be analyzed at component resolution time. This environment performs the service orchestration process, resulting in the generation of the SDL document (1a), which describes the set of components required for the service to run properly, in addition to a number of restrictions that will be used by the harmonizer for optimal component resolution into capabilities.

#### Execution Environment (2)

It is responsible for processing the SDL document and generating the graphical visualization of the service. This environment starts the process of component resolution, which is carried out by the Harmonizer.

#### Harmonizer (3)

Its main function is to make the matchmaking between the component to execute and a compatible capability (3a) from those available at the capability repository (3b). The aim of the Harmonizer is to select the best capability for each component grounded on different sources of information (user profile, customization options in components, context information, capability definition and so on). We use the Strategy pattern [10] to manage and apply different algorithm families to capability selection strategies.

#### Capability Access Middleware (4)

It performs capability discovery and invocation tasks and manages the events received, providing a uniform interface to the Harmonizer for data access (4b). Invocations are processed by some synchronous and asynchronous communication processors, which depend on the communication paradigm. We use the Command pattern [10] to encapsulate all the necessary information to process a sync/async invocation. To deal with asynchronous communication we have chosen to use the Proactor pattern [10], that uses the inversion control mechanism (in callback methods, 3c) to decouple application-independent asynchrony mechanisms from application-specific functionality. Callback methods are invoked when an event appears, such as a message arrival to the Access Middleware through a connection to a capability and perform application-specific processing. The component resolution process as well as the synchronous and asynchronous invocation management is further explained in Section 4.

An important requirement to be considered in environments with a large amount of heterogeneous capabilities is how to provide mechanisms for effective reuse of communication technologies during resource accessing. The Capability Access Middleware represents the fundamental level of reusability and follows the Acceptor/Connector pattern [10], which decouples the connection among tasks from the processing performed once the connection was carried out. This is achieved using various connection drivers (4c). In Section 5 we develop the key aspects of event and connection management.

In order to achieve flexible communication with smart devices the access middleware is able to change the communication paradigm in real time. The Communication Manager module (4d) receives information from the Invocation Manager and decides whether the paradigm change is appropriated, regarding the invocation frequency, the number of running services and the nature of the smart device. Section 7 describes the Communication Manager in detail.

An access middleware for mobile environments is characterized, from the ubiquitous computing point of view, by the large number of connections and disconnections that occur in a continuously changing environment and the appearance and disappearance of new capabilities. Proper management of resources is needed for efficient capability access. This management is facilitated by the use of the Monitor Object pattern [10] (4e) which synchronizes method execution to ensure that only one method runs within an object at a time. It also allows an object's methods to cooperatively schedule their execution sequences. Section 8 describes resource management in detail and the optimization algorithms we have defined for the Middleware.

## 5. Continuous Component Resolution in Mobile Environments

The harmonization subsystem provides a continuous component resolution process. The resolution is carried out using capabilities that are available in the user's current context. These capabilities are accessible in the own mobile device (e.g., GPS device), by proximity (e.g., printers, screens, *etc.*) or they are globally accessible, using telecommunication networks (e.g., 3G, GSM, *etc.*). Since these capabilities may disappear, the resolution process does not only take place at the beginning of each capability usage but also occurs when the Harmonizer determines that a change of capability is appropriate or necessary (e.g., a user with a GPS device enters an indoor environment). This subsystem also incorporates other advanced features, such as the suspension of running services and the detection of those new available capabilities that impeded a service execution.

The selection of the optimal capability for each component is done taking into account the user's preferences (e.g., higher priority for cheapest or closest devices) and component and capability descriptions, expressed using a XML language (see [4] for more details). A set of conditions can be defined to act as restrictions over a property, using comparison operators (*i.e.*, ==, >=, <=, =, !=). These conditions are converted to other query languages like SPARQL or SQL to perform the matching process. The usage of an XML language decouples the restriction representation from a specific storage and matching technology and enables to perform capability selection in devices with more limited computational resources.

The selection of the optimal capability for a specific component is a problem which depends on the current user's context, his preferences and the available capabilities. For example, price, proximity or the capability's underlying communication protocol are possible aspects which could be applied and combined in different forms for selection. Due to the existence of multiple applicable possibilities and combinations, a single selection algorithm could not be always applied and, if possible, it would be too difficult to extend by including new functionalities.

To avoid the previous problems, the Harmonizer applies the Strategy pattern, which is a design pattern that defines a common interface for a family of algorithms, allowing the applied algorithm to be interchangeable, independently of the element using it. Each capability selection strategy can be managed individually and, furthermore, applied by the Harmonizer depending on the current user's needs. In addition, the usage of this pattern allows for the inclusion of new selection strategies dynamically, in the form of plugins, without the need of redeploying the whole application in the mobile device. An application of the Strategy pattern to the domain of data sorting, which is closely related to the selection of the best available capability, is explained in [11].

**Figure 3.**
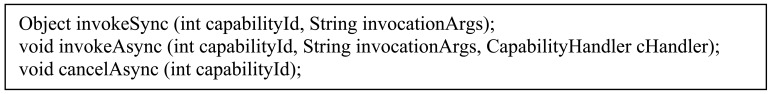
Capability Invocation API.

Once the resolution process has finished, the Harmonizer is responsible for transmitting any component invocation performed by the Execution Subsystem to the Capability Middleware and returning the execution results to the Result Rendering module (see Figure 2). The messages exchanged among the Execution, Harmonization and Capability Middleware subsystems are Java Objects, which encapsulate the transmitted parameters. The Invocation API (see Figure 3) contains generic methods for capability invocation, distinguishing between synchronous and asynchronous invocations.

**Figure 4.**
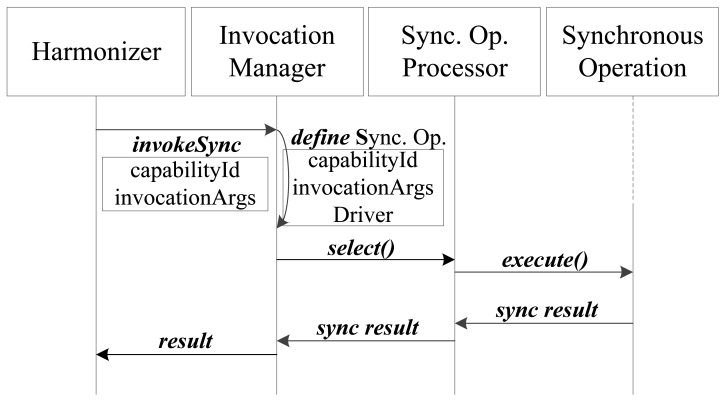
Interaction diagram of synchronous invocation.

The *invokeSync* method returns the result of the synchronous invocation through the Invocation Manager (see Figure 4). The *capabilityId* parameter indicates the selected capability and the *invocationArgs* parameter is a String array that contains the name of the method to be invoked and the parameters required for the method to run properly. This method returns a Java Object as a result, which is transmitted to the execution environment. After the Invocation Manager receives the synchronous access request, it creates a Synchronous Operation, designed by following the Command design pattern, which encapsulates all the necessary information to process the request (capability identification, *capabilityId*; arguments needed to perform the invocation, *invocationArgs*; driver identification, *driverId*) and defines an *execute()* method. This method performs the invocation request through a Driver, which controls the access technology, using *capabilityId* and *invocationArgs*. After that, the Invocation Manager selects a Synchronous Operation Processor to perform the Synchronous Operation in a new thread. There exist a limited number of Operation Processors, according to the number of Communication Paradigms that this middleware supports.

**Figure 5.**
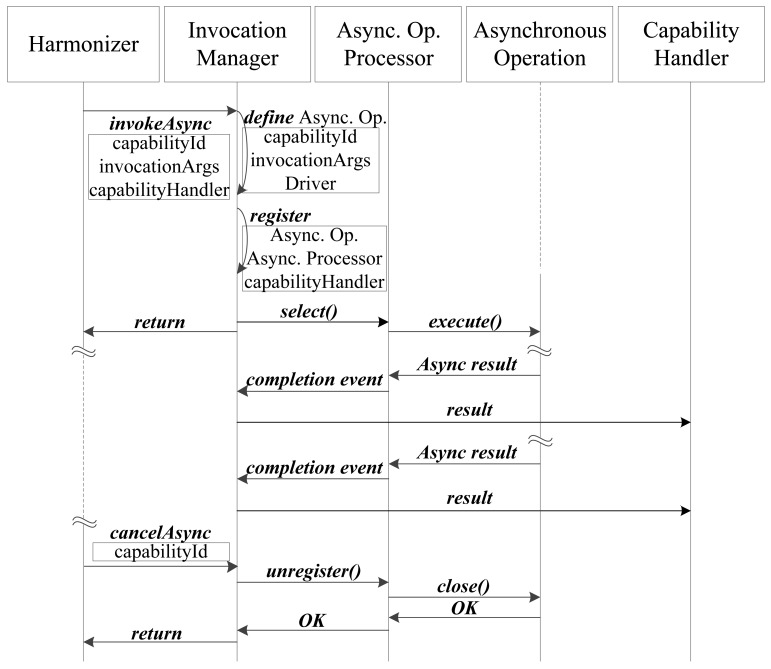
Interaction diagram of asynchronous invocation.

In the case of the asynchronous call (*invokeAsync* method), the common solution is to use a multi-threaded technique to perform operations in parallel (synchronous multi-threading). Every requested operation is executed in a thread that is scheduled by a manager. It is easy to write code for one thread, but the synchronization among many threads is a challenging task [12]. Nevertheless, in our work, the inversion mechanism provided by the Proactor pattern is used. The Proactor architecture pattern demultiplexes and dispatches completion events that are triggered by the completion of asynchronous operations. These completion events are dispatched to concrete service handlers that process them. Figure 5 shows the developed implementation of the Proactor pattern for asynchronous communication between the Harmonizer and the Capability Middleware subsystems.

The Harmonizer consumes the API provided by the Middleware and invokes the *invokeAsync (capabilityId, invocationArgs, capabilityHandler)* method, where *capablityHandler* is the reference to an object that can process the asynchronous result once it is received. The *invokeAsync* method is implemented by the Invocation Manager, which has two different roles: on one hand it defines the Asynchronous Operation as in the synchronous case and on the other hand registers the Asynchronous Operation with the *capabilityHandler* provided by the Harmonizer. Thus, once the asynchronous invocation is completed and the result is returned, the Invocation Manager can retrieve the Capability Handler from the registry and send it the result.

Once the registration is performed, the Invocation Manager selects an Asynchronous Operation Processor to perform the Asynchronous Operation in a new thread. When the operation finishes executing, a completion event is generated by the Asynchronous Operation Processor, which notifies the Invocation Manager. Then, the Invocation Manager dispatches to the associated capability handler, which processes the results of the asynchronous operation.

When the execution environment does not wish to receive asynchronous invocation results from capabilities, either because the service is over or the service execution logic no longer requires asynchronous access to data, the Harmonizer uses the *cancelAsync* API method, to cancel the event subscription. This invocation requests the Asynchronous Processor that is processing the Asynchronous Operation to terminate the connection to the capability. Once the connection has been completed, the Invocation Manager unregisters the Asynchronous Operation and returns a message indicating whether everything went well or not.

## 6. Communication Architecture

A middleware that provides a single communication paradigm could not cope with the variety of sensors, actuators, controllers and other devices that act as capabilities in our environment, making their use very limited. This middleware solution offers a set of communication paradigms ranging from the traditional synchronous model to different variations of the asynchronous model.

We define a *Synchronous/Asynchronous Operation Processor* as an entity chosen by the Invocation Manager to perform a sync/async operation. This operation may return an immediate result, as in the case of synchronous invocation or it may generate a series of events routed toward a Capability Handler, which is responsible for their processing. The way events containing invocation results are handled depends on the type of communication paradigm applied; therefore, there must be as many Operation Processors as Communication Paradigms are supported by the Middleware.

In the Capability Access Middleware we have implemented support for Request-Reply, QP2P and Publish-Subscribe communication models. The implications on the proposed architecture are described below:

- *Request-Reply*: the Operation Processor defined for this synchronous model runs directly the invocation operation, blocking the execution and awaiting the outcome, which is returned as a synchronous result. In order to avoid blocking problems on long-lasting requests, the Harmonizer controls the invocation requests using threads.
- *QP2P*: the Operation Processor, through a queue used for sending messages, has the possibility to handle asynchronous capability invocation in an independent way. In addition, it can also wait to receive some execution orders in order to make complex capability invocations. By having a queue for the receiving messages, the Operation Processor can return Completion Events composed of several responses. This is useful to send several responses received from the capability in a single message to the upper layers. For example, this paradigm is useful when accessing the Bluetooth pulse oximeter microX Medical RGB model [13], since the information it provides is composed of two messages containing the values of pulse and oxygen saturation. Instead, the capability handler for this device needs access to the two values at once. We consider the most correct way of dealing with this device is to wait to have the two values to produce a composite completion event.
- *Publish-Subscribe*: this paradigm provides Subscribers with the ability to express their interest in a topic or set of topics in order to be notified subsequently of any incoming events generated by a Publisher, which match the registered interest. This middleware integrates a topic-based publish-subscribe mechanism with the addition of an external service called Event Channel, which provides storage and management for subscriptions and efficient delivery of events. When the Operation Processor needs to subscribe to any capability, it creates an object of the class Topic with the information of the capability and an object of type Subscriber, which subscribes to the topic. The Subscriber object implements the method notify (Message m), which will receive messages published by the capability.

Because of the need to establish and maintain connections that use scarce resources in the mobile terminal (Bluetooth stack and ports), a Resource Controller Module has been incorporated to the proposed middleware for connection management, which optimizes connection duration and reduces data access delay. In the previous section, we described the usefulness of the connection drivers to decouple the invocation processing from the technology used for capability access. In order to implement this decoupling, we have used the Acceptor/Connector pattern, which defines two entities called Acceptor and Connector. The Acceptor is responsible for creating an endpoint that passively listens to connection requests in a particular address. The Connector connects to a remote Acceptor. In this pattern, there is an element called *ServiceHandler*, which provides a hook method that is called by an Acceptor or Connector to activate the application service when the connection is established. Once a Service Handler is completely initialized by an Acceptor or Connector factory it typically does not interact with these components any further.

Invocation drivers contain an Acceptor and Connector entities. The former listens to capability connection requests while the latter (that is the one used most often) makes requests over external capabilities. ServiceHandlers adapt and uniform the invocation result and deliver it to the Sync/Async Operation Processor for further processing.

**Figure 6.**
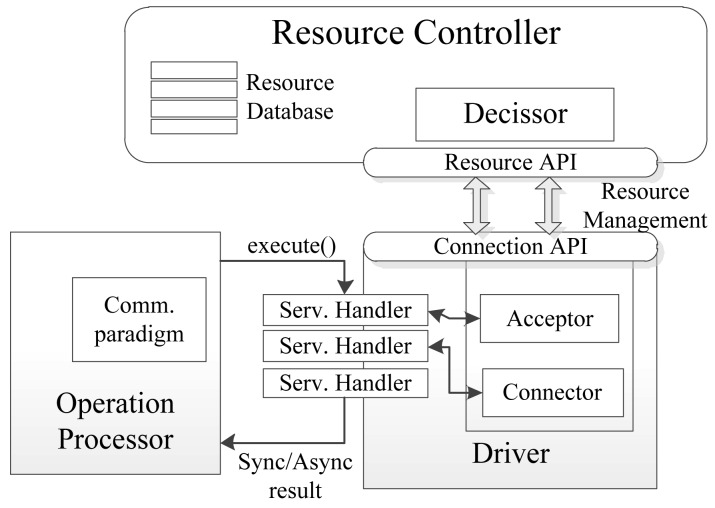
Internal driver communication.

Figure 6 shows an implementation of the communication between the Access Driver, the Operation Processor that executes it and the Resource Controller. In each driver there is a pool of ServiceHandlers, managing information from different types of capabilities. This design seeks to standardize data from heterogeneous devices so that can be recognized by Middleware's upper layers (Harmonizer and Execution subsystem). Between the drivers and the Resource Controller, two interfaces are defined, Resource API and Connection API, which exchange messages for controlling resources, an issue that we describe in the next section.

## 7. Communication with Smart Devices

The capabilities that are commonly accessed by the described middleware often restrict the communication paradigm used. For example, the Request-Reply paradigm determines the behavior of many devices and sensors that transmit the contained information when receiving a request message. Other devices also support asynchronous transmission, allowing them to send data sequentially, without needing to continuously receive request messages from consumer services [14]. This allows them to save resources, as they don't process request messages for each data to be transmitted. However, sending data without knowing their demand may not have an acceptable performance, since the device does not know a priori if there are other entities willing to consume the information it provides.

We call smart devices to those devices that support various communication paradigms, responding to synchronous and asynchronous information requests. In this section we describe how the push/pull model, deeply studied in the literature of wireless sensor networks [14,15], is integrated in our model with the support of the Request-Reply and the Publish-Subscribe communication paradigms.

Interoperability problems in communication (in terms of technologies, protocols and format of exchanged data) between the smart device and the middleware are outside the scope of the paper. Thus, we say that the use of a specific communication model is automatically performed by the smart device when it receives a data request in the language or protocol supported by the paradigm. The proposed middleware allows changes in the selected communication paradigm at two levels. At the service level, the change is produced by the alternated use of the *invokeSync* and the *invokeAsync* methods when calling the capability access middleware. At the communication level, it is produced by the choice of the correct service handler inside a communication driver. In this section we focus on the paradigm change at the communication level.

The middleware defined in this paper detects if the capability corresponds to a smart device through the discovery module, which accesses the capability description document and identifies all the needed parameters to invoke it. Flexible communication with smart devices (being able to change the communication paradigm on demand) reduces the number of transmissions between the communication middleware and the smart device.

### 7.1. Communication Paradigm Change

In this section we study under what conditions the paradigm change process is activated and how the capability access middleware supports the change in the communication model without affecting the upper level (communication with the harmonizer) and the communication quality with the device.

The use of a Publish-Subscribe communication model instead of the Request-reply model eliminates the request messages generated by the middleware. In contrast, the frequency of publication messages must be synchronized with the frequency of information request from the execution environments to avoid sending publications of data that will not be used or having delay or synchronization problems. Thus, the middleware request a subscription to the content generated by a smart device with a given publication rate. This rate will be accepted by the smart device if it has the necessary resources (the requested publication rate is lesser that the maximum sampling rate). To resolve synchronization problems between the middleware and the intelligent devices, the middleware is able to send back a subscription message with a new requested publication rate.

**Figure 7.**
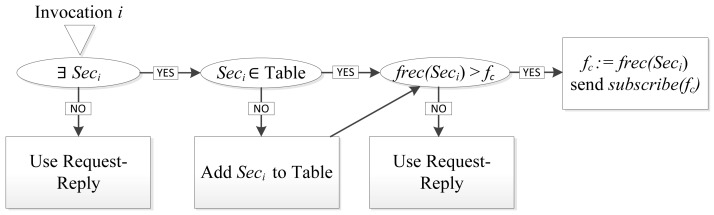
Communication paradigm selection process.

The proposed service model considers that the execution environment may be running different services that access the same intelligent device with different invocation rates or single invocations (e.g., the invocations produced by user interaction with interface elements such as buttons). The invocation manager maintains a table with the identifier of the smart capabilities and its request frequency (*f*). At the beginning of service execution, *f* is unknown, but it is learned after receiving a number (*n*) of invocation requests separated by an interval *T* = *1/f*. Due to the difficulty to deal with periodic invocations with different periods on the same capability, we have decided to give priority to the periodic invocation with higher frequency and use the Publish-Subscribe paradigm, whereas other periodic and single invocations will be performed by using Request-Reply. For each received invocation request, the invocation manager applies the process described in Figure 7.

If the received invocation is part of a sequence of invocations the process checks whether the sequence frequency is higher than the current frequency *f_c_*, which is currently used in Pub-Sub invocations. If the new frequency is higher the process assigns this frequency to the Pub-Sub invocations, replacing *f_c_* and sending a new Subscribe message with the new requested publication rate. Otherwise the invocation is done using Request-Reply.

Being I:= {*i_1_, i_2_*, …, *i_c_*} the set of invocations received by the middleware at the times *t_1_* to *t_c_*, and *T_12_* = *t_2_* − *t_1_*, in order to check if the invocation *i_c_ ∈* I, received in *t_c_*, corresponds to a sequence, the algorithm described in pseudocode in Algorithm 1 is applied. This algorithm checks if a received invocation corresponds to a sequence. To do that, it calculates the time differences between all the stored invocations and checks for periods between invocations that match *n* times.

**Algorithm 1.** Sequence check algorithm.1:i:= c − 1;2:calculate T_ic_;3:for 1 to n:4: if ∄ j ∈ {1,…, i−1} / T_ic_ = T_ji_;5: then i:= i − 1; goto 2;6: else save (j) in results; c:= i; i:= j;7:if results.length < n return false;8:else return true;

### 7.2. Architecture Implications

As indicated in Section 5, there exist a limited number of Operation Processors, according to the number of Communication Paradigms that this middleware supports. Therefore, in the Communication Paradigm change process, the original Sync/Async Operation Processor will be maintained, depending on the method invoked by the Harmonizer, invokeSync or invokeAsync.

Regarding the system architecture, the change of the communication paradigm in periodic invocation to smart capabilities can only be performed when the *invocationArgs* of the periodic invocations are the same. If not, these invocations cannot be considered as periodic and they are treated individually, as single invocations. Therefore, the considered periodic invocations generate the same Synchronous or Asynchronous Operation object, which contains the capability identifier *capabilityId*, the invocation arguments *invocationArgs*, and the *Driver* that will perform the invocation. This driver, specifically designed to access the smart capability, manages the paradigm change through the service handlers that it contains. These service handlers are adapted to the communication paradigms supported by the smart device.

The paradigm is chosen by the Communication Manager module, which consumes information from the Invocation Manager, regarding the number of invocations and the time in which they occur. The Communication Manager communicates with connection drivers via the Connection API, indicating which service handler should handle the invocation and the parameters to use (invocation period, subscription and unsubscription information).

**Figure 8.**
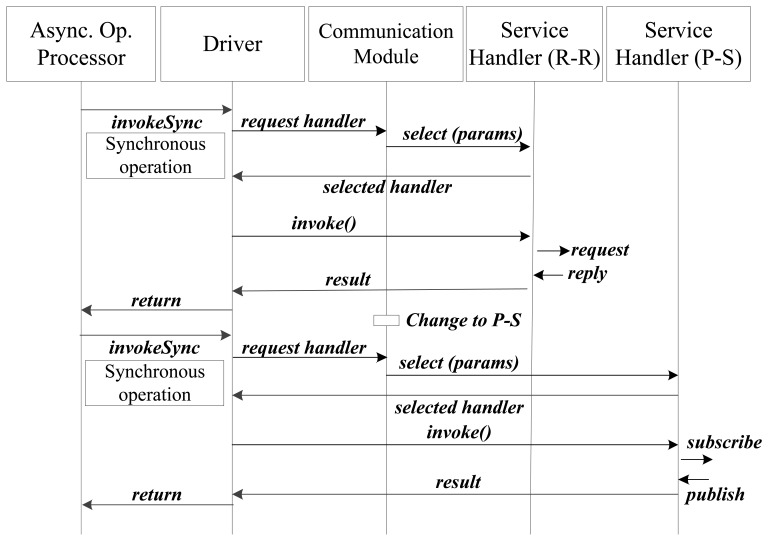
Interaction diagram of a Request-Reply to Publish-Subscribe paradigm change.

Figure 8 shows an interaction diagram of a Request-Reply to Publish-Subscribe paradigm change. The communication module, in the first invocation, selects the Service Handler that uses the Request-Reply paradigm. In the second invocation the Communication Module decides to change the paradigm to Publish-Subscribe. Therefore the communication module selects the P-S service handler and sends it some parameters such as requested publication frequency or whether a subscription or unsubscription is required.

After the last interaction described in the interaction diagram the Service Handler (P-S) will receive publications form the smart device without having to send the *subscribe* message again. Section 9 shows a validation of the paradigm change in a typical service execution scenario from Mobile Prosumer Environments.

## 8. Resource Management for Capability Discovery and Invocation

**Figure 9.**
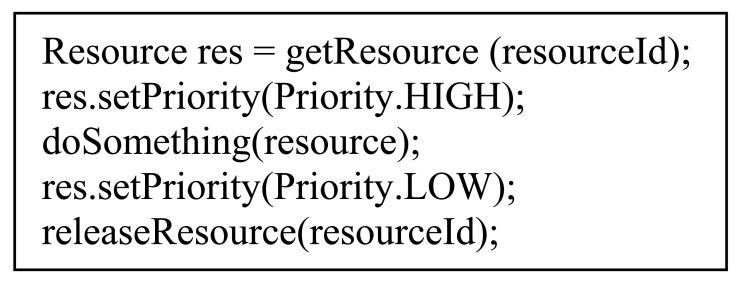
Resource request in drivers.

The environment described in our work defines capability access as a fundamental mechanism for a prosumer service since it enables to obtain the needed functionality at execution time. In the previous section we considered that these services request access to capabilities for repetitive invocations with a constant frequency. These invocations concurrently use resources of the mobile terminal that can be considered as limited (communication ports and Bluetooth stack). Therefore, we have defined mechanisms for resource management by using the Monitor Object concurrency pattern. This pattern synchronizes method execution to make sure that only one method is executed at a time. Thus, different drivers can concurrently attempt to access a common resource, but an internal mechanism will synchronize access to it, allowing access to one driver at a time. In Java, *synchronized* methods are used for this task. Figure 9 shows how a driver obtains a resource and uses it: first the driver should contact the Resource API for a Resource Object, then it establishes the priority to acquire the resource and, after using the resource, the driver releases it.

The Decissor module, in the Resource Controller, selects which invocation acquires the resource based on profiles. If the selected profile (by user preferences or depending on the capability type) seeks to reduce energy consumption in the Access Middleware, it will minimize the parameter $\bar{U_{C_{x}}}$. This parameter defines the average utilization rate of a resource for connections with an X capability, given that maintaining an open connection without being used increases battery consumption (from 6.6 mW in stand-by state to 69 mW in connected state for Bluetooth in the work of Cano *et al.* [16]). But if the objective is to minimize the invocation delay, the middleware adopts a profile that attempts to increase $\bar{U_{C_{x}}}$, so that connections are always active (see delay analysis in the validation section). We have implemented these two profiles with two algorithms called ESA (Energy Saving Algorithm) and SOA (Session Optimization Algorithm).

The ESA algorithm is simple: When the driver requests a resource to perform a capability connection, the Resource Controller blocks the request until the resource has become free. When the driver stops using the resource, it can invoke *setPriority(Priority.LOW)* or *releaseResource()* to indicate that it does not need this resource for a while.

The SOA algorithm is described in Algorithm 2. Be R_x_ a resource X, Q_Hx_ and Q_Lx_ priority and non-priority invocation queues that use R_x_, and I_Cy_ an invocation to Y capability, the Decissor applies the algorithm when it receives a resource request.

**Algorithm 2.** Pseudocode of Session Optimization Algorithm.(*I_C_y__, R_x_*) setPriority (Priority.HIGH)1:add to *Q_Hx_*2:if *R_x_* is used by *I_C_z__* with Priority.LOW then3: release(*R_x_*) from *I_C_z__*4: assign(*R_x_*) to first element of *Q_Hx_*5: wait() until *R_x_* is assigned to *I_C_y__*6: return(*I_C_y__, R_x_*) setPriority (Priority.LOW)1:add to *Q_Lx_*2:if *R_x_* is not used then3: assign(*R_x_*) to first element of *Q_Lx_*4: wait() until *R_x_* is assigned to *I_C_y__*5: return(*I_C_y__, R_x_*) releaseResource ()1: release(*R_x_*) from *I_C_y__*2: assign(*R_x_*) to first element of *Q_Hx_*

In order to release and assign resources, the Decissor interacts with the Connection API for communicating to drivers. Finally, there is also a thread that assigns R_x_ to the first element of Q_Hx_ and, if Q_Hx_ is empty, to the first element of Q_Lx_.

## 9. Validation

This work has been validated as part of a prototype implementation that consists of a Creation Environment, Execution Environment, Harmonizer [4] and Access Middleware, according to the mIO! project's architecture devised for mobile service provision. Section 9.1 describes the implementation of the Capability Access Middleware and the communication with smart devices whereas Section 9.2 presents a performance evaluation of the two algorithms for resource management.

### 9.1. Prototype Evaluation

The developed middleware follows the architecture described in Figure 2, integrating the Request-Reply, QP2P and Publish-Subscribe communication paradigms and the explained design patterns. The discovery module and some drivers that control capability access have also been developed, using REST, Bluetooth (RFCOMM and OBEX), SOAP and Java local access. For this proof of concept, we have tested access to Google Maps and Ovi Maps using REST, control of an UPnP / DLNA network hard drive (Model Media Iomega Home Network Drive) through SOAP and connection to a B600 FRWD heart rate monitor and a BT microX Medical RGB [13] pulse oximeter.

The capability access middleware has been implemented in Java ME, integrated with the Harmonization Subsystem and tested in a Nokia N97 and Nokia 5800 XpressMusic (O.S. Symbian S60 5° Ed) devices.

To evaluate the communication with smart devices the mobile terminal has been used to simulate a set of smart sensors that support the Request-Reply and Publish-Subscribe paradigms. We define a realistic service execution scenario in which there are three services running simultaneously and accessing the capability offered by a smart device as described in Table 3. The *n* parameter, used by the communication manager, indicates the amount of invocations with the same frequency that are needed to consider those invocations within the same sequence. The *δ* parameter enables to establish a margin of variation to recognize invocations within the same sequence. With the values of *δ* and *n* shown in this table no error was found in recognizing the invocation frequencies of Services 1, 2 and 3, although their invocation frequency is very close.

**Figure 10.**
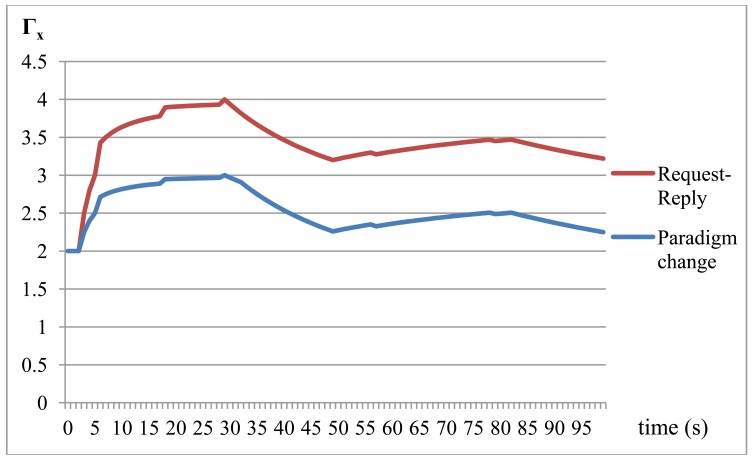
Transmission comparison in Service Execution Scenario.

Figure 10 represents the behavior of the communication middleware when receiving the invocations described in Table 3. Γ_x_ is the average number of transmissions performed by the communication middleware with the device that simulates the behavior of the smart services. The difference becomes noticeable once Service #2 starts, in the second 3.29, from which the application of the paradigm change model saves up to 25% of the transmissions. From 160 total invocations to the intelligent device, that is, 320 Request-Reply transmissions, the use of the paradigm change model reduces this number to 224, that is, a 70%.

### 9.2. Performance Comparison

As a proof of concept for resource management we present a performance evaluation of capability access using the internal Bluetooth capability (through JSR 82) of the mobile terminal that is executing the Capability Access Middleware. The aim of this study is to compare the behavior of the Resource Controller for each of the defined algorithms (ESA and SOA) in these two use cases:

Case #1: The system runs a service that accesses the Bluetooth resource every 6 seconds.
Case #2: The system runs two services accessing via Bluetooth to different capabilities periodically, with a frequency of 5 and 12 seconds.

In these cases we are not taking into account the discovery time and we assume that the Bluetooth service accessed is known. If Bluetooth capabilities were unknown, the Discovery module (which also uses the Bluetooth resource) would be needed. Thus, discovery is modeled as another capability that accesses resources for the Resource Controller's point of view.

**Figure 11.**
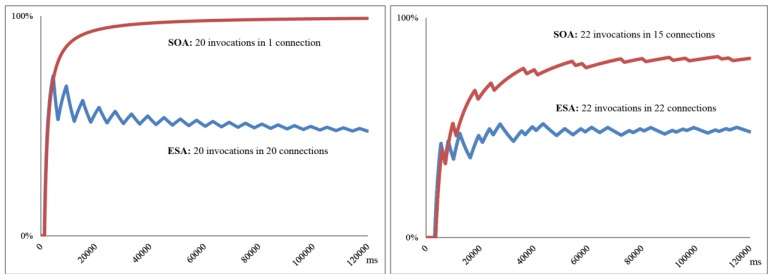
Average resource utilization for Bluetooth in Case #1 (left) and Case #2 (right).

As mentioned in Section 8 and for the Bluetooth case, the ESA algorithm purpose is to optimize power consumption by minimizing the utilization of the Bluetooth resource. On the other hand, the SOA algorithm purpose is to minimize the Bluetooth capability access whilst maintaining the connection with the Bluetooth resource as long as possible. We found that the average delay for data access using the studied Bluetooth capability (BT microX Medical RGB pulse oximeter) corresponds to 3,953 ms (0.2 standard deviation) and 1,988 ms (0.3 standard deviation) if the connection was already established before. Figure 11 analyzes the value of $\bar{U_{C}}$ (Average resource usage rate for connections with studied capabilities) for both use cases and ESA and SOA algorithms, knowing that a low value of $\bar{U_{C}}$ optimizes power consumption, while a value of $\bar{U_{C}}$ close to 100% determine a lower access delay.

These figures show that the difference between the two algorithms in terms of channel usage for connections is clear. In Case #1 with ESA algorithm the average resource usage for connections tends to 100% as time passes, due to the fact that the Middleware creates a single connection, which holds every invocation (20 invocations in 1 connection for 120 seconds). In the case of SOA, the resource is used just to receive the capability data, which corresponds to about 50% utilization. In Case #2 (which is a more realistic behavior for a multi-execution environment), the difference in values, although significant, is not as extreme; since in both cases it is necessary to make disconnections (22 *versus* 15) to release the resource in order to be used by other capabilities.

## 10. Related Work

This section concentrates on reviewing previous work on communication middleware in continuous service execution environments. Continuous service execution, studied as service roaming in related work [17,18], enables the consumption of services in a mobile environment. Services are only valid in a specific scope, becoming useless where the user moves outside it. Context changes are monitored to check for valid scopes during the execution, triggering the search for other compatible services when current scope changes. Ontologies and service roaming are used in conjunction in [19]. However, none of these approaches takes into account the particular characteristics of micro-services in prosumer environments, where users create their own services, which are provided to others. For detailed study of service provision in mobile prosumer environments the authors refer to their previous work [4].

There are many types of communication middlewares, Message Oriented (MoM), Remote Procedure Call (RPC), Object Request Broker (ORB) and even Service-oriented Architecture Middlewares [20]. While traditional middleware platforms typically employ synchronous, RPC-style client/server interactions, MoMs provide asynchronous, peer-to-peer style interactions, leading to a more loosely coupled architecture which is more adequate for dynamic mobile environments [21]. Related work in communication middleware for dynamic environments (such as the mobile prosumer environment) provide flexibility and reconfiguration to their systems in two levels, in the architectural and development model level and in the communication support level.

In the architecture level, some research has enhanced CORBA-based middleware to become flexible, customizable and lightweight. For example, UIC [22], based on dynamicTAO [23], an extension of CORBA, provides a reflective architecture that detects the presence of a remote device and loads at runtime the adequate communication driver to manage the connection. To achieve adaptability and flexibility, in addition to use reflective techniques, they are also employed some real time refactoring techniques for models [24], detecting antipatterns and ill-structures, and also for the programming level, as in the work of Binley *et al.* [25], who use AspectJ to refactor OOP (Object Oriented Programming) programs into equivalent AOP (Aspect Oriented Programming) programs. Other works [26] opt to generate architecture-level programs, providing flexibility by modeling of variation points and design patterns at various architecture levels. The adaptability to requirements of new environments or locations is also tacked in the literature. AmbientSoaML [27] introduces ambients (bounded places where computation occurs) in Service oriented architecture Modeling Language (SoaML) to extend this metamodel with mobility concerns and decoupling them from the business logic. The concept of ambient is also introduced in the work of Ali *et al.* [28] in the form of connectors that offer mobility services to architectural elements and coordinate element boundaries. The notions of adaptability and separation of concerns of these two approaches are also addressed in our work by using communication paradigms and design patterns respectively. However, the scope of our work is different, as we focus on a programming perspective and the works of Ali *et al.* in the metamodel design.

With regard to the communication support level we highlight the works in reconfigurable communication middleware. The PLA middleware [29] has been designed as a flexible and lightweight middleware for ubiquitous computing, aimed for mobile terminals. The main difference with our proposal, from the point of view of software engineering, is that they combine minimal fine-grained components and use a mixin layer approach [30] to tailor the architecture to fit in a specific scenario. MUSIC [31] also extends a generic middleware, which seamlessly supports component-based and service-based configurations. The functionality provided by a component can be dynamically configured to adapt the framework to different environments. In our work does not extend or particularize a basic middleware core but it develops a complete solution which focuses in mobile prosumer environments and is designed specifically for it.

Integrating communication paradigms in Access Middleware has been tackled in [21], proposing an architecture which supports the traditional synchronous model and different variations of the so-called asynchronous models. Other works, as GREEN [32], focus in the concept of reconfiguration in continuous execution environments, and provide a reconfigurable middleware (according to application requirements and context information) that supports publish-subscribe interaction types (topic-based, content-based and location-based) but only for one communication paradigm.

In ubiquitous computing environments, devices might not be connected at all times. Several proposals take this into account and support that devices enter and leave networks on an *ad hoc* basis. This behavior can be modeled by using P2P networks [33], in which devices are peers and communicate via *ad hoc* protocols. To locate these devices, some content-based techniques are used, such as Distributed Hash Tables (DHT). Other works [34] introduce the concept of Ubiquitous Consumer Wireless World (UCWW), where the consumer changes its access network provider to use available and suitable services in a continuous way, following the “always best connected and best served” paradigm.

Related works in resource management are divided between those that provide mechanisms for overload prevention, that is, provide message prioritization and load balancing [35], and those which rely on adaptation mechanisms that change the access protocol or session QoS parameters. Regarding the latter, MUM [36] proposes a dynamic and flexible middleware to support continuous services to mobile users by migrating the session state in response to user movements during service provisioning. It also integrates some sync/async client/server paradigms but it focuses in session management and preservation rather than device access or architectural issues. In [6], a Session Initiation Protocol middleware is provided for session management, which also provides resource reservation and QoS management for user services. However, resource reservation is done at the session level, through SDP (Session Description Protocol). Our work supports session management by the Harmonizer and the connection-oriented communication paradigms (request-reply and publish-subscribe), and also enables physical resource reservation, as in the Bluetooth case, analyzed in detail in Section 8.

## 11. Conclusions and Future Work

This paper proposes a solution for enabling flexible and continuous capability invocation in ubiquitous environments. This solution is focused in mobile prosumer environments, in which the user is the center of the environment and his mobile phone is the gateway for interacting with the surrounding capabilities. The requirements imposed by this environment determine the existence of three processes: Orchestration, Resolution and Capability Invocation. Focusing on the latter, our main contribution in this work is specified by the definition and implementation of an architecture for a communication middleware and its integration with other dependent subsystems, such the Harmonizer, as part of an overall architecture for the mIO! project. This architecture has been developed following the design patterns Strategy, Command, Proactor, Acceptor/Connector and Monitor Object [10], in order to meet the requirements of strategy-based resolution, low coupling, asynchronism, reusability and efficient resource management respectively.

The communication paradigms that are present in the Access Middleware (Request-Reply, QP2P and Publish-Subscribe) allow it to cope with the heterogeneity of sensors, actuators, controllers and other devices in the environment. The middleware implementation fulfills the task of decoupling capability access from the selection of the optimal capability and from the processing of the generated information. In order to deal with the so called smart devices, the developed middleware supports the automatic communication paradigm change. We focus in the push/pull model, describing the change from the Request-reply to the Publish-Subscribe paradigm and *vice versa*. In the evaluation section we determine that using the described paradigm change model and algorithm we can save up to 25% of the transmissions between the communication middleware and the smart devices.

Finally, we have made a contribution related to the management of limited resources in the mobile terminal that performs capability access by comparing the performance of two algorithms for Bluetooth access in terms of energy consumption and data access delay. This leads to the conclusion that the Access Middleware must be able to decide which algorithm to use depending on the parameter to optimize (delay or consumption), which will be given by user preferences or provided by contextual information.

In the same way that the Harmonizer incorporates different decision strategies for component resolution, as future work we plan to extend the communications middleware architecture to improve the intelligent selection of the optimal communication paradigm. Also, we consider the concept of dynamic service deployment [37] in the form of OSGi bundles, applied to automatic driver deployment in the communication middleware so that we can extend our current work to new and heterogeneous capabilities.

## Figures and Tables

**Table 1.** Communication paradigms and features.

**Paradigm**	**Coordination mechanism**	**Notification model**	**Connection Orientation**	**Connection initiated by**	**Design requirements**
Request/Reply	Synchronous	Synchronous	✓	Consumer (Middleware)	Client-server model
QP2P	Asynchronous	Synchronous	✗	Producer/Consumer	Messages are retrieved in a predefined order
Tuple Spaces	Asynchronous	Synchronous	✗	✗	Intermediated by a tuple space service
Publish-Subscribe	Asynchronous	Asynchronous	✓	Event Channel Service	Event channel service must be external

**Table 2.** Requirements, patterns and design implications.

**Requisite**	**Associated pattern**	**Application**	**Design implications**
Capability selection strategies	Strategy	Harmonizer	Inclusion of selection strategies in the form of plugins. Plugin management.
Low coupling in arch. modules	Command	Sync/Async operations	Definition of Async/Sync operation and processors.
Asynchronous communication	Proactor	Invocation and discovery API	Inversion control mechanism: Callback and hook method definition in harmonizer.
Capability Access reusability	Acceptor/Connector	Communication paradigms	Pool of service handlers for each connection driver. Bidirectional communication in drivers.
Efficient resource management	Monitor Object	Shared resource controller	Concurrency management in limited resources

**Table 3.** Service Execution Scenario.

**Services**	**Start Time (s)**	**Duration (s)**	**Invocation Frequency (Inv/s)**
Serv. #1	0.0	100	1
Serv. #2	3.39	27.6	1.087
Serv. #3	50.32	33	0.909
**Communication Manager parameters**		δ = 10 ms	*n* = 3 invocations
